# (μ-*trans*-1,2-Di-4-pyridylethyl­ene-κ^2^ *N*:*N*′)bis­[bis­(*N*,*N*-diethyl­dithio­carbamato-κ^2^ *S*,*S*′)zinc(II)] chloro­form solvate

**DOI:** 10.1107/S1600536809044237

**Published:** 2009-10-31

**Authors:** Hadi D. Arman, Pavel Poplaukhin, Edward R. T. Tiekink

**Affiliations:** a Department of Chemistry, The University of Texas at San Antonio, One UTSA Circle, San Antonio, Texas 78249-0698, USA; b Chemical Abstracts Service, 2540 Olentangy River Rd, Columbus, Ohio 43202, USA; c Department of Chemistry, University of Malaya, 50603 Kuala Lumpur, Malaysia

## Abstract

The dinuclear title solvate, [Zn_2_(C_5_H_10_NS_2_)_4_(C_12_H_10_N_2_)]·CHCl_3_, features two five-coordinate Zn atoms; both coordination polyhedra are distorted, but one has an NS_4_ donor set approximating to a square pyramid (with the N atom in the apical site), while the other is closer to a ZnNS_4_ trigonal-bipyramidal arrangement (with the N atom in an equatorial site). In both cases, the Zn^II^ atom is chelated by two *S*,*S*′-bidentate dithiol­ate ligands. In the crystal, the chloro­form solvent mol­ecules reside in cavities defined by the dinuclear species and are held in place *via* C—H⋯S contacts.

## Related literature

For background to supra­molecular polymers of zinc 1,1-dithiol­ates, see: Lai *et al.* (2002 ▶); Chen *et al.* (2006 ▶); Benson *et al.* (2007 ▶). For a related structure and the synthesis, see: Lai & Tiekink (2003 ▶). For additional geometrical analysis, see: Addison *et al.* (1984 ▶).
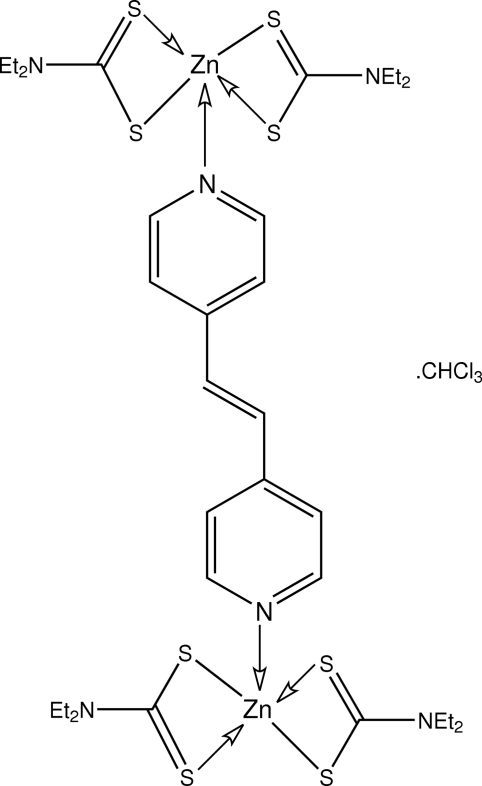


## Experimental

### 

#### Crystal data

[Zn_2_(C_5_H_10_NS_2_)_4_(C_12_H_10_N_2_)]·CHCl_3_
*M* *_r_* = 1025.37
Orthorhombic, 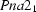

*a* = 17.443 (3) Å
*b* = 15.739 (3) Å
*c* = 16.823 (3) Å
*V* = 4618.5 (14) Å^3^
*Z* = 4
Mo *K*α radiation
μ = 1.61 mm^−1^
*T* = 98 K
0.35 × 0.21 × 0.09 mm

#### Data collection

Rigaku AFC12K/SATURN724 diffractometer
Absorption correction: multi-scan (*ABSCOR*; Higashi, 1995 ▶) *T*_min_ = 0.824, *T*_max_ = 1
36476 measured reflections
9260 independent reflections
8995 reflections with *I* > 2σ(*I*)
*R*_int_ = 0.050

#### Refinement

*R*[*F* ^2^ > 2σ(*F* ^2^)] = 0.034
*wR*(*F* ^2^) = 0.075
*S* = 1.07
9260 reflections
477 parameters
1 restraint
H-atom parameters constrained
Δρ_max_ = 0.83 e Å^−3^
Δρ_min_ = −0.40 e Å^−3^
Absolute structure: Flack (1983 ▶), 3766 Friedel pairs
Flack parameter: −0.004 (8)

### 

Data collection: *CrystalClear* (Rigaku/MSC, 2005 ▶); cell refinement: *CrystalClear*; data reduction: *CrystalClear*; program(s) used to solve structure: *SHELXS97* (Sheldrick, 2008 ▶); program(s) used to refine structure: *SHELXL97* (Sheldrick, 2008 ▶); molecular graphics: *DIAMOND* (Brandenburg, 2006 ▶); software used to prepare material for publication: *SHELXL97*.

## Supplementary Material

Crystal structure: contains datablocks global, I. DOI: 10.1107/S1600536809044237/hb5173sup1.cif

Structure factors: contains datablocks I. DOI: 10.1107/S1600536809044237/hb5173Isup2.hkl

Additional supplementary materials:  crystallographic information; 3D view; checkCIF report

## Figures and Tables

**Table 1:** Selected bond lengths (Å)

Zn1—N5	2.069 (3)
Zn1—S1	2.3567 (9)
Zn1—S3	2.3659 (9)
Zn1—S2	2.5629 (9)
Zn1—S4	2.5654 (9)
Zn2—N6	2.075 (3)
Zn2—S7	2.3381 (10)
Zn2—S5	2.3526 (9)
Zn2—S6	2.4934 (10)
Zn2—S8	2.6575 (9)

**Table 2:** Hydrogen-bond geometry (Å, °)

*D*—H⋯*A*	*D*—H	H⋯*A*	*D*⋯*A*	*D*—H⋯*A*
C33—H33⋯S8^i^	1.00	2.72	3.598 (4)	149

Symmetry code: (i) 
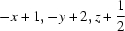
.

**Fig. 1.**
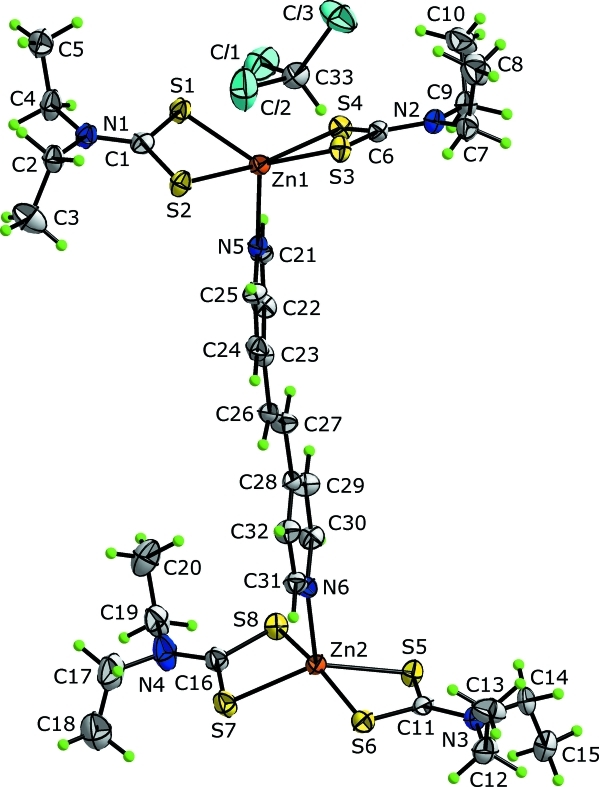
The molcular structure of (I) showing displacement ellipsoids at the 70% probability level.

## supplementary crystallographic information

### Comment

The title compound (I) was prepared in connection with crystal engineering studies of zinc(II) 1,1-dithiolates (Lai *et al.*, 2002; Chen *et al.*, 2006; Benson *et al.* 2007). The dinuclear compound features two five coordinate Zn atoms, each being coordinated by two dithiocarbamate ligands and a pyridine-N atom. The dithiocarbamate ligands are chelating but form disparate Zn—S bond distances that range from 2.3381 (10) to 2.6575 (9) Å. The coordination geometries for the Zn1 and Zn2 atoms are each based on a NS_4_ donor set but are distinct. Thus, the geometries for the Zn1 and Zn2 atoms are distorted towards trigonal bipyramidal (TB) and square pyramidal (SP), respectively. This is quantified by the values of τ = 0.56 and 0.36, respectively, compared with the ideal values of 1.0 and 0.0 for TB and SP, respectively (Addison *et al.*, 1984).

The most closely related structure available for comparison is the diethyldithiocarbamate analogue of (I) which was co-crystallized with a *trans*-1,2-bis(4-pyridyl)ethylene molecule (Lai & Tiekink, 2003) rather than a solvent chloroform molecule as for (I). In the former, the range of Zn—S bond distances was considerably narrower, *i.e.* 2.4100 (10) to 2.4914 (11) Å, and the coordination geometry for Zn (the dinuclear molecule is centrosymmetric) was close to SP (τ = 0.13).

The solvent chlorofrom molecules in (I) reside in cavities defined by the dinuclear molecules and are held in place by C—H···S interactions, Table 1,

### Experimental

Compound (I) was prepared by following a standard literature procedure (Lai & Tiekink, 2003) whereby two equivalents of Zn(S_2_CNEt_2_)_2_ were added to *trans*-1,2-bis(4-pyridyl)ethylene. Crystals were obtained from slow evaporation of a chloroform solution. When heated, the crystals turned opaque at 349–351 K and melted at 401–403 K.

### Refinement

The H atoms were geometrically placed (C—H = 0.95–0.99 Å) and refined as riding with *U*_iso_(H) = 1.2–1.5*U*_eq_(C).

### Crystal data

**Table:**

[Zn_2_(C_5_H_10_NS_2_)_4_(C_12_H_10_N_2_)]·CHCl_3_	*F*(000) = 2120
*M**_r_* = 1025.37	*D*_x_ = 1.475 Mg m^−^^3^
Orthorhombic, *P**n**a*2_1_	Mo *K*α radiation, λ = 0.71069 Å
Hall symbol: P 2c -2n	Cell parameters from 19833 reflections
*a* = 17.443 (3) Å	θ = 2.1–40.8°
*b* = 15.739 (3) Å	µ = 1.61 mm^−^^1^
*c* = 16.823 (3) Å	*T* = 98 K
*V* = 4618.5 (14) Å^3^	Block, yellow
*Z* = 4	0.35 × 0.21 × 0.09 mm

### Data collection

**Table:**

Rigaku AFC12K/SATURN724 diffractometer	9260 independent reflections
Radiation source: fine-focus sealed tube	8995 reflections with *I* > 2σ(*I*)
graphite	*R*_int_ = 0.050
ω scans	θ_max_ = 27.5°, θ_min_ = 2.1°
Absorption correction: multi-scan (*ABSCOR*; Higashi, 1995)	*h* = −22→22
*T*_min_ = 0.824, *T*_max_ = 1	*k* = −20→20
36476 measured reflections	*l* = −17→21

### Refinement

**Table:**

Refinement on *F*^2^	Secondary atom site location: difference Fourier map
Least-squares matrix: full	Hydrogen site location: inferred from neighbouring sites
*R*[*F*^2^ > 2σ(*F*^2^)] = 0.034	H-atom parameters constrained
*wR*(*F*^2^) = 0.075	*w* = 1/[σ^2^(*F*_o_^2^) + (0.0266*P*)^2^ + 3.3574*P*] where *P* = (*F*_o_^2^ + 2*F*_c_^2^)/3
*S* = 1.07	(Δ/σ)_max_ = 0.001
9260 reflections	Δρ_max_ = 0.83 e Å^−^^3^
477 parameters	Δρ_min_ = −0.40 e Å^−^^3^
1 restraint	Absolute structure: Flack (1983), 3766 Friedel pairs
Primary atom site location: structure-invariant direct methods	Flack parameter: −0.004 (8)

### Special details

**Table:**

Geometry. All s.u.'s (except the s.u. in the dihedral angle between two l.s. planes) are estimated using the full covariance matrix. The cell s.u.'s are taken into account individually in the estimation of s.u.'s in distances, angles and torsion angles; correlations between s.u.'s in cell parameters are only used when they are defined by crystal symmetry. An approximate (isotropic) treatment of cell s.u.'s is used for estimating s.u.'s involving l.s. planes.
Refinement. Refinement of *F*^2^ against ALL reflections. The weighted *R*-factor *wR* and goodness of fit *S* are based on *F*^2^, conventional *R*-factors *R* are based on *F*, with *F* set to zero for negative *F*^2^. The threshold expression of *F*^2^ > 2σ(*F*^2^) is used only for calculating *R*-factors(gt) *etc*. and is not relevant to the choice of reflections for refinement. *R*-factors based on *F*^2^ are statistically about twice as large as those based on *F*, and *R*- factors based on ALL data will be even larger.

### Fractional atomic coordinates and isotropic or equivalent isotropic displacement parameters (Å^2^)

**Table:**

	*x*	*y*	*z*	*U*_iso_*/*U*_eq_	
Zn1	0.48561 (2)	0.46529 (2)	0.25986 (2)	0.01771 (8)	
Zn2	0.52421 (2)	1.04699 (2)	−0.32727 (2)	0.01811 (8)	
S1	0.37402 (5)	0.47688 (5)	0.33808 (5)	0.02142 (17)	
S2	0.39216 (5)	0.35268 (5)	0.20769 (5)	0.02060 (16)	
S3	0.60161 (4)	0.38777 (5)	0.26719 (5)	0.01975 (16)	
S4	0.58224 (5)	0.55975 (5)	0.33339 (5)	0.02023 (16)	
S5	0.64756 (4)	1.10741 (5)	−0.32528 (6)	0.02142 (16)	
S6	0.59961 (5)	0.96317 (5)	−0.42580 (5)	0.02180 (17)	
S7	0.40173 (5)	1.06415 (6)	−0.38140 (6)	0.02441 (18)	
S8	0.45491 (5)	1.17094 (5)	−0.24764 (5)	0.02131 (16)	
N1	0.25911 (16)	0.38216 (17)	0.28033 (17)	0.0202 (6)	
N2	0.71730 (16)	0.47956 (17)	0.32773 (17)	0.0202 (6)	
N3	0.74526 (16)	1.01190 (18)	−0.40881 (17)	0.0220 (6)	
N4	0.30787 (18)	1.1551 (2)	−0.2899 (2)	0.0353 (8)	
N5	0.47859 (15)	0.54237 (15)	0.16051 (18)	0.0165 (6)	
N6	0.51719 (15)	0.95913 (15)	−0.2356 (2)	0.0179 (5)	
C1	0.33315 (19)	0.40127 (18)	0.2750 (2)	0.0193 (6)	
C2	0.22382 (19)	0.3145 (2)	0.2327 (2)	0.0214 (7)	
H2A	0.1822	0.2877	0.2639	0.026*	
H2B	0.2627	0.2704	0.2212	0.026*	
C3	0.1911 (3)	0.3475 (2)	0.1547 (2)	0.0349 (9)	
H3A	0.1549	0.3937	0.1655	0.052*	
H3B	0.1644	0.3013	0.1271	0.052*	
H3C	0.2329	0.3688	0.1212	0.052*	
C4	0.2053 (2)	0.4269 (2)	0.3333 (2)	0.0259 (7)	
H4A	0.1555	0.4337	0.3059	0.031*	
H4B	0.2256	0.4842	0.3452	0.031*	
C5	0.1932 (2)	0.3788 (3)	0.4108 (2)	0.0343 (9)	
H5A	0.1758	0.3209	0.3992	0.051*	
H5B	0.1543	0.4081	0.4428	0.051*	
H5C	0.2415	0.3766	0.4404	0.051*	
C6	0.64211 (18)	0.4769 (2)	0.3119 (2)	0.0182 (6)	
C7	0.7699 (2)	0.4104 (2)	0.3059 (2)	0.0254 (7)	
H7A	0.8193	0.4351	0.2886	0.030*	
H7B	0.7480	0.3788	0.2603	0.030*	
C8	0.7845 (2)	0.3483 (3)	0.3742 (3)	0.0358 (9)	
H8A	0.8133	0.3771	0.4164	0.054*	
H8B	0.8143	0.2998	0.3546	0.054*	
H8C	0.7354	0.3282	0.3954	0.054*	
C9	0.7508 (2)	0.5538 (2)	0.3680 (2)	0.0243 (7)	
H9A	0.7268	0.6059	0.3464	0.029*	
H9B	0.8062	0.5562	0.3555	0.029*	
C10	0.7411 (3)	0.5535 (3)	0.4577 (2)	0.0343 (9)	
H10A	0.6868	0.5466	0.4709	0.051*	
H10B	0.7599	0.6074	0.4796	0.051*	
H10C	0.7705	0.5064	0.4806	0.051*	
C11	0.67278 (19)	1.0258 (2)	−0.3888 (2)	0.0185 (6)	
C12	0.7677 (2)	0.9474 (2)	−0.4679 (2)	0.0278 (8)	
H12A	0.8156	0.9656	−0.4944	0.033*	
H12B	0.7272	0.9430	−0.5090	0.033*	
C13	0.7798 (2)	0.8609 (2)	−0.4302 (2)	0.0340 (9)	
H13A	0.8204	0.8647	−0.3900	0.051*	
H13B	0.7948	0.8199	−0.4712	0.051*	
H13C	0.7321	0.8421	−0.4049	0.051*	
C14	0.80844 (19)	1.0610 (2)	−0.3729 (2)	0.0274 (8)	
H14A	0.8547	1.0247	−0.3698	0.033*	
H14B	0.7940	1.0773	−0.3180	0.033*	
C15	0.8274 (2)	1.1405 (2)	−0.4197 (2)	0.0322 (8)	
H15A	0.8483	1.1245	−0.4716	0.048*	
H15B	0.8654	1.1741	−0.3905	0.048*	
H15C	0.7807	1.1742	−0.4272	0.048*	
C16	0.38029 (19)	1.1340 (2)	−0.3040 (2)	0.0212 (7)	
C17	0.2414 (2)	1.1077 (3)	−0.3288 (3)	0.0414 (10)	
H17A	0.2569	1.0485	−0.3405	0.050*	
H17B	0.1971	1.1061	−0.2921	0.050*	
C18	0.2196 (3)	1.1518 (3)	−0.4038 (3)	0.0451 (11)	
H18A	0.2003	1.2087	−0.3914	0.068*	
H18B	0.1795	1.1191	−0.4309	0.068*	
H18C	0.2646	1.1564	−0.4384	0.068*	
C19	0.2882 (2)	1.2217 (2)	−0.2313 (2)	0.0301 (8)	
H19A	0.2416	1.2521	−0.2493	0.036*	
H19B	0.3306	1.2633	−0.2283	0.036*	
C20	0.2740 (2)	1.1845 (3)	−0.1493 (3)	0.0383 (10)	
H20A	0.2313	1.1440	−0.1519	0.058*	
H20B	0.2611	1.2302	−0.1121	0.058*	
H20C	0.3202	1.1551	−0.1309	0.058*	
C21	0.47506 (18)	0.62720 (19)	0.1688 (2)	0.0187 (6)	
H21	0.4723	0.6503	0.2209	0.022*	
C22	0.47521 (19)	0.6821 (2)	0.1050 (2)	0.0199 (7)	
H22	0.4718	0.7417	0.1136	0.024*	
C23	0.48044 (18)	0.6501 (2)	0.0279 (2)	0.0185 (6)	
C24	0.48146 (19)	0.5615 (2)	0.0190 (2)	0.0209 (7)	
H24	0.4826	0.5366	−0.0324	0.025*	
C25	0.48072 (18)	0.5108 (2)	0.0864 (2)	0.0199 (7)	
H25	0.4818	0.4509	0.0798	0.024*	
C26	0.48606 (19)	0.7044 (2)	−0.0429 (2)	0.0197 (7)	
H26	0.4815	0.6784	−0.0937	0.024*	
C27	0.4971 (2)	0.7877 (2)	−0.0400 (2)	0.0212 (7)	
H27	0.5004	0.8129	0.0113	0.025*	
C28	0.50485 (19)	0.8446 (2)	−0.10882 (19)	0.0170 (6)	
C29	0.5127 (2)	0.9317 (2)	−0.0959 (2)	0.0233 (7)	
H29	0.5140	0.9536	−0.0433	0.028*	
C30	0.51845 (19)	0.9857 (2)	−0.1599 (2)	0.0208 (7)	
H30	0.5236	1.0448	−0.1500	0.025*	
C31	0.51125 (18)	0.87512 (19)	−0.2482 (2)	0.0197 (6)	
H31	0.5119	0.8549	−0.3014	0.024*	
C32	0.5043 (2)	0.8172 (2)	−0.1877 (2)	0.0212 (7)	
H32	0.4992	0.7585	−0.1995	0.025*	
C33	0.4971 (2)	0.7614 (3)	0.4505 (2)	0.0313 (8)	
H33	0.5306	0.7820	0.4063	0.038*	
Cl1	0.44088 (7)	0.84716 (6)	0.48451 (7)	0.0425 (2)	
Cl2	0.43669 (6)	0.68037 (8)	0.41320 (8)	0.0503 (3)	
Cl3	0.55577 (7)	0.72255 (6)	0.52699 (7)	0.0431 (3)	

### Atomic displacement parameters (Å^2^)

**Table:**

	*U*^11^	*U*^22^	*U*^33^	*U*^12^	*U*^13^	*U*^23^
Zn1	0.01558 (16)	0.02010 (16)	0.01744 (19)	−0.00015 (13)	0.00102 (15)	0.00359 (15)
Zn2	0.01462 (16)	0.02072 (17)	0.01900 (19)	−0.00070 (13)	0.00125 (15)	0.00434 (15)
S1	0.0177 (4)	0.0264 (4)	0.0202 (4)	−0.0051 (3)	0.0021 (3)	−0.0059 (3)
S2	0.0192 (4)	0.0200 (4)	0.0226 (4)	−0.0006 (3)	0.0035 (3)	−0.0026 (3)
S3	0.0168 (3)	0.0178 (3)	0.0247 (4)	−0.0009 (3)	0.0000 (3)	0.0002 (3)
S4	0.0200 (4)	0.0203 (4)	0.0204 (4)	0.0006 (3)	−0.0014 (3)	−0.0009 (3)
S5	0.0165 (3)	0.0225 (3)	0.0253 (4)	−0.0024 (3)	0.0019 (3)	−0.0017 (3)
S6	0.0208 (4)	0.0244 (4)	0.0202 (4)	−0.0017 (3)	0.0016 (3)	−0.0008 (3)
S7	0.0187 (4)	0.0318 (4)	0.0227 (4)	0.0054 (3)	−0.0028 (3)	−0.0070 (4)
S8	0.0191 (4)	0.0207 (3)	0.0241 (4)	−0.0002 (3)	−0.0003 (3)	−0.0009 (3)
N1	0.0179 (13)	0.0221 (13)	0.0206 (15)	−0.0028 (10)	0.0016 (11)	−0.0041 (11)
N2	0.0175 (13)	0.0220 (13)	0.0210 (15)	−0.0029 (10)	0.0000 (11)	0.0011 (11)
N3	0.0175 (14)	0.0279 (14)	0.0205 (15)	0.0030 (11)	0.0009 (11)	0.0035 (12)
N4	0.0213 (16)	0.0437 (19)	0.041 (2)	0.0091 (14)	−0.0024 (14)	−0.0157 (16)
N5	0.0147 (12)	0.0169 (12)	0.0178 (16)	−0.0021 (9)	−0.0002 (10)	0.0002 (10)
N6	0.0172 (12)	0.0188 (12)	0.0179 (14)	0.0014 (9)	−0.0004 (11)	0.0018 (11)
C1	0.0212 (15)	0.0160 (13)	0.0206 (17)	−0.0023 (11)	−0.0013 (12)	0.0031 (12)
C2	0.0191 (15)	0.0206 (15)	0.0246 (18)	−0.0026 (12)	−0.0009 (13)	−0.0035 (13)
C3	0.043 (2)	0.0285 (18)	0.034 (2)	0.0029 (16)	−0.0098 (18)	−0.0050 (16)
C4	0.0174 (16)	0.0299 (17)	0.030 (2)	−0.0014 (13)	0.0040 (14)	−0.0107 (15)
C5	0.0229 (18)	0.054 (2)	0.026 (2)	−0.0052 (17)	0.0018 (15)	−0.0114 (18)
C6	0.0171 (15)	0.0217 (14)	0.0159 (16)	−0.0040 (12)	0.0006 (12)	0.0020 (12)
C7	0.0163 (16)	0.0306 (17)	0.029 (2)	0.0020 (13)	0.0011 (14)	−0.0009 (15)
C8	0.031 (2)	0.035 (2)	0.041 (3)	0.0033 (16)	−0.0037 (18)	0.0049 (18)
C9	0.0196 (17)	0.0286 (17)	0.0249 (19)	−0.0063 (13)	−0.0022 (14)	0.0006 (14)
C10	0.039 (2)	0.040 (2)	0.024 (2)	−0.0037 (18)	−0.0075 (17)	−0.0049 (17)
C11	0.0173 (15)	0.0226 (15)	0.0156 (15)	0.0035 (12)	0.0038 (12)	0.0048 (12)
C12	0.0229 (18)	0.0343 (19)	0.026 (2)	0.0108 (14)	0.0054 (15)	0.0014 (16)
C13	0.041 (2)	0.037 (2)	0.024 (2)	0.0156 (17)	−0.0002 (17)	−0.0029 (16)
C14	0.0120 (15)	0.042 (2)	0.028 (2)	0.0027 (14)	−0.0019 (13)	0.0044 (16)
C15	0.0198 (17)	0.042 (2)	0.035 (2)	−0.0045 (15)	0.0030 (16)	0.0036 (18)
C16	0.0192 (16)	0.0223 (15)	0.0222 (19)	0.0017 (12)	−0.0007 (13)	−0.0006 (13)
C17	0.0250 (19)	0.052 (2)	0.047 (3)	0.0083 (17)	0.007 (2)	−0.008 (2)
C18	0.034 (2)	0.050 (3)	0.051 (3)	0.0033 (19)	−0.002 (2)	−0.006 (2)
C19	0.0235 (17)	0.0352 (18)	0.032 (2)	0.0109 (14)	−0.0013 (15)	−0.0086 (17)
C20	0.035 (2)	0.035 (2)	0.046 (3)	−0.0018 (17)	0.0116 (19)	−0.0060 (19)
C21	0.0184 (14)	0.0202 (14)	0.0174 (16)	0.0011 (11)	−0.0007 (13)	−0.0028 (13)
C22	0.0213 (16)	0.0183 (14)	0.0201 (18)	0.0009 (12)	−0.0032 (13)	−0.0008 (13)
C23	0.0150 (15)	0.0225 (16)	0.0181 (17)	−0.0012 (12)	−0.0005 (12)	0.0012 (13)
C24	0.0198 (16)	0.0209 (15)	0.0218 (18)	−0.0029 (12)	−0.0012 (13)	−0.0035 (14)
C25	0.0210 (16)	0.0191 (15)	0.0197 (17)	−0.0008 (12)	0.0002 (13)	0.0026 (13)
C26	0.0222 (17)	0.0220 (15)	0.0149 (16)	−0.0010 (12)	−0.0016 (12)	0.0019 (13)
C27	0.0250 (17)	0.0225 (15)	0.0161 (16)	0.0000 (13)	0.0006 (13)	−0.0021 (13)
C28	0.0166 (15)	0.0185 (14)	0.0159 (16)	0.0014 (12)	0.0021 (12)	0.0023 (12)
C29	0.0290 (19)	0.0216 (16)	0.0192 (18)	0.0011 (13)	0.0012 (14)	0.0007 (14)
C30	0.0227 (16)	0.0171 (15)	0.0227 (18)	−0.0007 (11)	0.0005 (13)	0.0013 (13)
C31	0.0217 (15)	0.0206 (14)	0.0167 (17)	−0.0012 (12)	0.0002 (13)	−0.0006 (13)
C32	0.0262 (17)	0.0178 (14)	0.0196 (17)	−0.0029 (12)	0.0005 (14)	−0.0003 (13)
C33	0.0268 (19)	0.040 (2)	0.027 (2)	−0.0002 (16)	0.0031 (16)	0.0057 (17)
Cl1	0.0430 (6)	0.0343 (5)	0.0501 (7)	0.0026 (4)	0.0076 (5)	0.0064 (5)
Cl2	0.0277 (5)	0.0651 (7)	0.0579 (7)	−0.0034 (5)	0.0009 (5)	−0.0286 (6)
Cl3	0.0532 (7)	0.0335 (5)	0.0426 (6)	−0.0029 (4)	−0.0174 (5)	0.0094 (4)

### Geometric parameters (Å, °)

**Table:**

Zn1—N5	2.069 (3)	C9—H9B	0.9900
Zn1—S1	2.3567 (9)	C10—H10A	0.9800
Zn1—S3	2.3659 (9)	C10—H10B	0.9800
Zn1—S2	2.5629 (9)	C10—H10C	0.9800
Zn1—S4	2.5654 (9)	C12—C13	1.517 (5)
Zn2—N6	2.075 (3)	C12—H12A	0.9900
Zn2—S7	2.3381 (10)	C12—H12B	0.9900
Zn2—S5	2.3526 (9)	C13—H13A	0.9800
Zn2—S6	2.4934 (10)	C13—H13B	0.9800
Zn2—S8	2.6575 (9)	C13—H13C	0.9800
S1—C1	1.746 (3)	C14—C15	1.514 (5)
S2—C1	1.711 (3)	C14—H14A	0.9900
S3—C6	1.741 (3)	C14—H14B	0.9900
S4—C6	1.709 (3)	C15—H15A	0.9800
S5—C11	1.728 (3)	C15—H15B	0.9800
S6—C11	1.728 (3)	C15—H15C	0.9800
S7—C16	1.745 (3)	C17—C18	1.490 (7)
S8—C16	1.712 (3)	C17—H17A	0.9900
N1—C1	1.329 (4)	C17—H17B	0.9900
N1—C2	1.468 (4)	C18—H18A	0.9800
N1—C4	1.473 (4)	C18—H18B	0.9800
N2—C6	1.339 (4)	C18—H18C	0.9800
N2—C7	1.470 (4)	C19—C20	1.519 (6)
N2—C9	1.472 (4)	C19—H19A	0.9900
N3—C11	1.326 (4)	C19—H19B	0.9900
N3—C14	1.476 (4)	C20—H20A	0.9800
N3—C12	1.473 (5)	C20—H20B	0.9800
N4—C16	1.327 (4)	C20—H20C	0.9800
N4—C19	1.479 (5)	C21—C22	1.377 (5)
N4—C17	1.525 (5)	C21—H21	0.9500
N5—C25	1.343 (5)	C22—C23	1.396 (5)
N5—C21	1.344 (4)	C22—H22	0.9500
N6—C30	1.341 (5)	C23—C24	1.402 (4)
N6—C31	1.343 (4)	C23—C26	1.470 (5)
C2—C3	1.523 (5)	C24—C25	1.386 (5)
C2—H2A	0.9900	C24—H24	0.9500
C2—H2B	0.9900	C25—H25	0.9500
C3—H3A	0.9800	C26—C27	1.326 (4)
C3—H3B	0.9800	C26—H26	0.9500
C3—H3C	0.9800	C27—C28	1.470 (5)
C4—C5	1.522 (6)	C27—H27	0.9500
C4—H4A	0.9900	C28—C29	1.395 (5)
C4—H4B	0.9900	C28—C32	1.395 (5)
C5—H5A	0.9800	C29—C30	1.375 (5)
C5—H5B	0.9800	C29—H29	0.9500
C5—H5C	0.9800	C30—H30	0.9500
C7—C8	1.530 (5)	C31—C32	1.372 (5)
C7—H7A	0.9900	C31—H31	0.9500
C7—H7B	0.9900	C32—H32	0.9500
C8—H8A	0.9800	C33—Cl3	1.754 (4)
C8—H8B	0.9800	C33—Cl1	1.764 (4)
C8—H8C	0.9800	C33—Cl2	1.769 (4)
C9—C10	1.519 (5)	C33—H33	1.0000
C9—H9A	0.9900		
			
N5—Zn1—S1	110.90 (8)	H10A—C10—H10C	109.5
N5—Zn1—S3	113.27 (8)	H10B—C10—H10C	109.5
S1—Zn1—S3	135.82 (4)	N3—C11—S6	121.3 (3)
N5—Zn1—S2	95.23 (8)	N3—C11—S5	121.4 (3)
S1—Zn1—S2	73.70 (3)	S6—C11—S5	117.29 (19)
S3—Zn1—S2	101.84 (3)	N3—C12—C13	111.9 (3)
N5—Zn1—S4	95.08 (7)	N3—C12—H12A	109.2
S1—Zn1—S4	103.21 (3)	C13—C12—H12A	109.2
S3—Zn1—S4	73.25 (3)	N3—C12—H12B	109.2
S2—Zn1—S4	169.66 (3)	C13—C12—H12B	109.2
N6—Zn2—S7	108.20 (8)	H12A—C12—H12B	107.9
N6—Zn2—S5	108.23 (8)	C12—C13—H13A	109.5
S7—Zn2—S5	142.61 (4)	C12—C13—H13B	109.5
N6—Zn2—S6	99.94 (8)	H13A—C13—H13B	109.5
S7—Zn2—S6	106.51 (3)	C12—C13—H13C	109.5
S5—Zn2—S6	74.98 (3)	H13A—C13—H13C	109.5
N6—Zn2—S8	95.04 (8)	H13B—C13—H13C	109.5
S7—Zn2—S8	72.29 (3)	N3—C14—C15	112.5 (3)
S5—Zn2—S8	96.44 (3)	N3—C14—H14A	109.1
S6—Zn2—S8	164.46 (3)	C15—C14—H14A	109.1
C1—S1—Zn1	86.85 (12)	N3—C14—H14B	109.1
C1—S2—Zn1	81.19 (11)	C15—C14—H14B	109.1
C6—S3—Zn1	87.36 (11)	H14A—C14—H14B	107.8
C6—S4—Zn1	81.79 (11)	C14—C15—H15A	109.5
C11—S5—Zn2	85.61 (11)	C14—C15—H15B	109.5
C11—S6—Zn2	81.30 (11)	H15A—C15—H15B	109.5
C16—S7—Zn2	88.75 (12)	C14—C15—H15C	109.5
C16—S8—Zn2	79.48 (12)	H15A—C15—H15C	109.5
C1—N1—C2	122.3 (3)	H15B—C15—H15C	109.5
C1—N1—C4	123.5 (3)	N4—C16—S8	122.7 (3)
C2—N1—C4	114.2 (3)	N4—C16—S7	119.7 (3)
C6—N2—C7	122.5 (3)	S8—C16—S7	117.67 (19)
C6—N2—C9	120.4 (3)	C18—C17—N4	109.2 (4)
C7—N2—C9	117.1 (3)	C18—C17—H17A	109.8
C11—N3—C14	121.5 (3)	N4—C17—H17A	109.8
C11—N3—C12	122.5 (3)	C18—C17—H17B	109.8
C14—N3—C12	116.1 (3)	N4—C17—H17B	109.8
C16—N4—C19	121.1 (3)	H17A—C17—H17B	108.3
C16—N4—C17	121.6 (3)	C17—C18—H18A	109.5
C19—N4—C17	117.1 (3)	C17—C18—H18B	109.5
C25—N5—C21	117.7 (3)	H18A—C18—H18B	109.5
C25—N5—Zn1	122.1 (2)	C17—C18—H18C	109.5
C21—N5—Zn1	120.1 (2)	H18A—C18—H18C	109.5
C30—N6—C31	117.2 (3)	H18B—C18—H18C	109.5
C30—N6—Zn2	119.8 (2)	N4—C19—C20	111.7 (3)
C31—N6—Zn2	123.0 (3)	N4—C19—H19A	109.3
N1—C1—S2	121.9 (3)	C20—C19—H19A	109.3
N1—C1—S1	120.6 (3)	N4—C19—H19B	109.3
S2—C1—S1	117.47 (19)	C20—C19—H19B	109.3
N1—C2—C3	112.4 (3)	H19A—C19—H19B	107.9
N1—C2—H2A	109.1	C19—C20—H20A	109.5
C3—C2—H2A	109.1	C19—C20—H20B	109.5
N1—C2—H2B	109.1	H20A—C20—H20B	109.5
C3—C2—H2B	109.1	C19—C20—H20C	109.5
H2A—C2—H2B	107.9	H20A—C20—H20C	109.5
C2—C3—H3A	109.5	H20B—C20—H20C	109.5
C2—C3—H3B	109.5	N5—C21—C22	122.9 (3)
H3A—C3—H3B	109.5	N5—C21—H21	118.6
C2—C3—H3C	109.5	C22—C21—H21	118.6
H3A—C3—H3C	109.5	C21—C22—C23	119.8 (3)
H3B—C3—H3C	109.5	C21—C22—H22	120.1
N1—C4—C5	111.7 (3)	C23—C22—H22	120.1
N1—C4—H4A	109.3	C22—C23—C24	117.4 (3)
C5—C4—H4A	109.3	C22—C23—C26	123.2 (3)
N1—C4—H4B	109.3	C24—C23—C26	119.4 (3)
C5—C4—H4B	109.3	C25—C24—C23	119.0 (3)
H4A—C4—H4B	107.9	C25—C24—H24	120.5
C4—C5—H5A	109.5	C23—C24—H24	120.5
C4—C5—H5B	109.5	N5—C25—C24	123.1 (3)
H5A—C5—H5B	109.5	N5—C25—H25	118.4
C4—C5—H5C	109.5	C24—C25—H25	118.4
H5A—C5—H5C	109.5	C27—C26—C23	123.7 (3)
H5B—C5—H5C	109.5	C27—C26—H26	118.2
N2—C6—S4	122.2 (2)	C23—C26—H26	118.2
N2—C6—S3	120.6 (2)	C26—C27—C28	125.9 (3)
S4—C6—S3	117.27 (19)	C26—C27—H27	117.1
N2—C7—C8	112.9 (3)	C28—C27—H27	117.1
N2—C7—H7A	109.0	C29—C28—C32	116.9 (3)
C8—C7—H7A	109.0	C29—C28—C27	119.0 (3)
N2—C7—H7B	109.0	C32—C28—C27	124.1 (3)
C8—C7—H7B	109.0	C30—C29—C28	119.6 (3)
H7A—C7—H7B	107.8	C30—C29—H29	120.2
C7—C8—H8A	109.5	C28—C29—H29	120.2
C7—C8—H8B	109.5	N6—C30—C29	123.3 (3)
H8A—C8—H8B	109.5	N6—C30—H30	118.3
C7—C8—H8C	109.5	C29—C30—H30	118.3
H8A—C8—H8C	109.5	N6—C31—C32	123.0 (3)
H8B—C8—H8C	109.5	N6—C31—H31	118.5
N2—C9—C10	114.3 (3)	C32—C31—H31	118.5
N2—C9—H9A	108.7	C31—C32—C28	120.0 (3)
C10—C9—H9A	108.7	C31—C32—H32	120.0
N2—C9—H9B	108.7	C28—C32—H32	120.0
C10—C9—H9B	108.7	Cl3—C33—Cl1	110.7 (2)
H9A—C9—H9B	107.6	Cl3—C33—Cl2	110.9 (2)
C9—C10—H10A	109.5	Cl1—C33—Cl2	109.6 (2)
C9—C10—H10B	109.5	Cl3—C33—H33	108.5
H10A—C10—H10B	109.5	Cl1—C33—H33	108.5
C9—C10—H10C	109.5	Cl2—C33—H33	108.5
			
N5—Zn1—S1—C1	84.10 (13)	C9—N2—C6—S4	2.7 (4)
S3—Zn1—S1—C1	−95.67 (11)	C7—N2—C6—S3	2.5 (4)
S2—Zn1—S1—C1	−5.33 (11)	C9—N2—C6—S3	−177.9 (2)
S4—Zn1—S1—C1	−175.12 (11)	Zn1—S4—C6—N2	174.3 (3)
N5—Zn1—S2—C1	−104.77 (13)	Zn1—S4—C6—S3	−5.19 (17)
S1—Zn1—S2—C1	5.50 (11)	Zn1—S3—C6—N2	−173.9 (3)
S3—Zn1—S2—C1	140.11 (11)	Zn1—S3—C6—S4	5.58 (18)
S4—Zn1—S2—C1	79.5 (2)	C6—N2—C7—C8	−96.2 (4)
N5—Zn1—S3—C6	84.91 (14)	C9—N2—C7—C8	84.2 (4)
S1—Zn1—S3—C6	−95.33 (12)	C6—N2—C9—C10	81.3 (4)
S2—Zn1—S3—C6	−174.04 (11)	C7—N2—C9—C10	−99.1 (4)
S4—Zn1—S3—C6	−3.45 (11)	C14—N3—C11—S6	−175.4 (2)
N5—Zn1—S4—C6	−109.25 (13)	C12—N3—C11—S6	4.8 (4)
S1—Zn1—S4—C6	137.86 (11)	C14—N3—C11—S5	4.9 (4)
S3—Zn1—S4—C6	3.54 (11)	C12—N3—C11—S5	−174.8 (2)
S2—Zn1—S4—C6	66.5 (2)	Zn2—S6—C11—N3	172.1 (3)
N6—Zn2—S5—C11	90.19 (14)	Zn2—S6—C11—S5	−8.27 (16)
S7—Zn2—S5—C11	−103.21 (12)	Zn2—S5—C11—N3	−171.6 (3)
S6—Zn2—S5—C11	−5.53 (11)	Zn2—S5—C11—S6	8.69 (17)
S8—Zn2—S5—C11	−172.33 (11)	C11—N3—C12—C13	−88.1 (4)
N6—Zn2—S6—C11	−100.79 (13)	C14—N3—C12—C13	92.1 (4)
S7—Zn2—S6—C11	146.70 (11)	C11—N3—C14—C15	−90.3 (4)
S5—Zn2—S6—C11	5.58 (11)	C12—N3—C14—C15	89.5 (4)
S8—Zn2—S6—C11	63.49 (16)	C19—N4—C16—S8	8.8 (5)
N6—Zn2—S7—C16	81.52 (14)	C17—N4—C16—S8	−166.8 (3)
S5—Zn2—S7—C16	−85.08 (13)	C19—N4—C16—S7	−172.7 (3)
S6—Zn2—S7—C16	−171.80 (12)	C17—N4—C16—S7	11.7 (5)
S8—Zn2—S7—C16	−8.02 (11)	Zn2—S8—C16—N4	166.5 (3)
N6—Zn2—S8—C16	−99.21 (14)	Zn2—S8—C16—S7	−12.03 (17)
S7—Zn2—S8—C16	8.31 (12)	Zn2—S7—C16—N4	−165.1 (3)
S5—Zn2—S8—C16	151.76 (12)	Zn2—S7—C16—S8	13.47 (19)
S6—Zn2—S8—C16	96.33 (16)	C16—N4—C17—C18	−92.5 (5)
S1—Zn1—N5—C25	−118.7 (2)	C19—N4—C17—C18	91.7 (5)
S3—Zn1—N5—C25	61.1 (2)	C16—N4—C19—C20	−92.2 (5)
S2—Zn1—N5—C25	−44.2 (2)	C17—N4—C19—C20	83.6 (4)
S4—Zn1—N5—C25	135.0 (2)	C25—N5—C21—C22	−1.4 (5)
S1—Zn1—N5—C21	64.2 (2)	Zn1—N5—C21—C22	175.8 (2)
S3—Zn1—N5—C21	−116.0 (2)	N5—C21—C22—C23	−1.1 (5)
S2—Zn1—N5—C21	138.7 (2)	C21—C22—C23—C24	3.1 (5)
S4—Zn1—N5—C21	−42.0 (2)	C21—C22—C23—C26	−175.9 (3)
S7—Zn2—N6—C30	−103.6 (2)	C22—C23—C24—C25	−2.8 (5)
S5—Zn2—N6—C30	67.9 (2)	C26—C23—C24—C25	176.2 (3)
S6—Zn2—N6—C30	145.2 (2)	C21—N5—C25—C24	1.7 (5)
S8—Zn2—N6—C30	−30.6 (2)	Zn1—N5—C25—C24	−175.4 (3)
S7—Zn2—N6—C31	76.4 (3)	C23—C24—C25—N5	0.4 (5)
S5—Zn2—N6—C31	−112.2 (2)	C22—C23—C26—C27	9.5 (5)
S6—Zn2—N6—C31	−34.8 (3)	C24—C23—C26—C27	−169.5 (3)
S8—Zn2—N6—C31	149.4 (2)	C23—C26—C27—C28	178.6 (3)
C2—N1—C1—S2	4.0 (4)	C26—C27—C28—C29	177.0 (4)
C4—N1—C1—S2	−175.4 (3)	C26—C27—C28—C32	−2.5 (6)
C2—N1—C1—S1	−175.2 (2)	C32—C28—C29—C30	0.8 (5)
C4—N1—C1—S1	5.4 (5)	C27—C28—C29—C30	−178.7 (3)
Zn1—S2—C1—N1	172.7 (3)	C31—N6—C30—C29	−1.1 (5)
Zn1—S2—C1—S1	−8.05 (16)	Zn2—N6—C30—C29	178.8 (3)
Zn1—S1—C1—N1	−172.1 (3)	C28—C29—C30—N6	−0.2 (5)
Zn1—S1—C1—S2	8.67 (17)	C30—N6—C31—C32	1.9 (5)
C1—N1—C2—C3	−91.9 (4)	Zn2—N6—C31—C32	−178.0 (3)
C4—N1—C2—C3	87.6 (4)	N6—C31—C32—C28	−1.3 (5)
C1—N1—C4—C5	−97.5 (4)	C29—C28—C32—C31	−0.1 (5)
C2—N1—C4—C5	83.0 (4)	C27—C28—C32—C31	179.4 (3)
C7—N2—C6—S4	−176.9 (3)		

### Hydrogen-bond geometry (Å, °)

**Table:**

*D*—H···*A*	*D*—H	H···*A*	*D*···*A*	*D*—H···*A*
C33—H33···S8^i^	1.00	2.72	3.598 (4)	149

Symmetry codes: (i) −*x*+1, −*y*+2, *z*+1/2.
